# National Insurance, Hospital Saturday and Insured Persons

**Published:** 1913-04-26

**Authors:** 


### The Hospital

The Workers' Newspaper of Administrative Medicine and Institutional Life, Administration, National Insurance and Health.

No. 1399, Vol. LIV. SATURDAY, APRIL 26, 1913. PRICE ONE PENNY.

### NATIONAL INSURANCE, HOSPITAL SATURDAY AND INSURED PERSONS.

The Hon. Harry Lawson, M.P., who is treasurer of the Hospital Saturday Fund, made a most statesmanlike and interesting speech at the thirty-eighth annual meeting of the Hospital Saturday Fund at the Mansion House on the 19th inst. Mr. Lawson yields to no one in his zeal and practical work on behalf of the hospitals and the sick. All workers for charity, and especially for hospitals, owe a great debt of gratitude to the *Daily Telegraph*, of which Mr. Lawson is the responsible editor, for the large amount of space devoted to these subjects and the excellent reports which that paper now invariably contains. Nothing is too small, no field of human suffering too remote, to gain publicity on its merits, should opportunity offer, in the columns of the *Daily Telegraph*. It follows that Mr. Harry Lawson has won for himself a place in hospital affairs, and his opinion deservedly carries weight and attracts attention. For this reason all hospital workers should make a point of obtaining a copy of the *Daily Telegraph* of the 21st instant and reading the speech to which we refer. Mr. Lawson was careful to point out at the commencement that, in spite of the National Insurance Act, serious illness and dangerous accidents, whether they came by the hand of man or, in the old phrase, by the hand of God, were just as much uninsurable risks to-day as they ever were. For this reason the Hospital Saturday Fund, the regnant principle of which is self-insurance, is just as necessary to-day as it was before the National Insurance Act became effective. These facts make it doubly hard for voluntary hospitals, which, in consequence of the Act, have had the special work cast upon them materially increased, and they have further to meet a growing demand on the part of the whole community, due to the great development in the extent of medical supervision and advice now prevalent everywhere. No worker, whatever may be the field of his or her labour, can preserve his maximum earning power without the ever-efficient and magnificent work of the voluntary hospitals. Yet the serious maladies and accidents to life, where hospital care and treatment are a paramount necessity, are still uninsurable risks, and it is a matter of ordinary prudence for the people of all classes to recognise the fact and to support such institutions with ever-increasing liberality, for in them alone is the highest skill and up-to-date experience available for the people at large. When the National Insurance Act first came into effective being, and the voluntary hospitals had to lay down a rule that they could not continue to receive as patients persons suffering only from such ailments as could be treated by a private practitioner, the fear arose that insured persons might be shut out altogether from hospital benefits. Speaking to the working classes throughout the metropolis, and as treasurer of the Hospital Saturday Fund, Mr. Lawson wisely urged that contributors to this Fund should allow these matters to be settled in an amicable and sensible fashion by those who represent the Fund on the one hand and those who represent the hospitals on the other. Both sides, after all, wished and meant to work together for the common good.

One great point which Mr. Lawson made was the enormous development in scientific treatment and the progress in medical and surgical methods which the hospitals now provide. These developments apply equally to public as to private health, the adequate maintenance of which rested upon the maximum of efficiency and the ability to take advantage of all the discoveries and appliances of science to secure the best results. It was incumbent upon every individual in these days of advanced education, where knowledge was placed within the reach of all classes of the community, to resolve to arm himself with new courage and new knowledge to enable all to live better and to live longer. Workers for the Hospital Saturday Fund and an army of workers for the hospitals, and especially for the League of Mercy, are fully aware of the truth and force of the principles just enunciated. This is really the secret of the great and continued success of the Voluntary Hospitals.

Mr. Lawson did not say anything on the financial aspects which underlie many of the principles which he so wisely advocated. The Government have announced in the House of Commons that they already have in preparation a Bill to amend the National Insurance Act. One great question which presses urgently for wise and generous consideration on the part of the Government is the remission of the duty on legacies, and the granting of power by Parliament to the Local Government Board under adequate safeguards, whilst preserving the voluntary system, to enable grants to be made to voluntary hospitals and institutions for buildings, and to strengthen the present system of National Insurance by enactments which will make it possible to secure and even to extend and improve the existing hospital accommodation, without the aid of which no system of National Insurance can continue sound in the financial sense. We have on more than one occasion formulated a practical plan of *pro rata* payments for insured persons needing in-patient treatment at the voluntary hospitals, whereby the expenditure on National Insurance will be largely diminished and the voluntary system of hospital support will not only be upheld but strengthened. Speaking from fulness of knowledge, we can say with confidence that not only several important members of the Government, but the men who know most about these subjects, have determined to place this question of hospital treatment upon the strongest possible basis by instituting a system of co-operation and *pro rata* payments which must contribute to the permanent improvement of the whole of our health service and to the perpetuation of the voluntary principle. So the British system of hospitals and public health would become the best in the world.

